# Badgers remain fearless in the face of simulated wolf presence near their setts

**DOI:** 10.1002/ece3.10654

**Published:** 2024-01-04

**Authors:** Tom A. Diserens, Marcin Churski, Jakub W. Bubnicki, Andrzej Zalewski, Marcin Brzeziński, Dries P. J. Kuijper

**Affiliations:** ^1^ Mammal Research Institute Polish Academy of Sciences Białowieża Poland; ^2^ Faculty of Biology University of Warsaw Warsaw Poland

**Keywords:** European badger, fossoriality, grey wolf, landscape of fear, large carnivore ecology, perceived risk, playback experiment

## Abstract

Many mesocarnivores are fossorial and use burrow systems to avoid predators. But fossorial animals cannot stay safely underground forever; they must also risk emerging overground to forage and find mates. To make this trade‐off effectively and maximise their own fitness, it is imperative they assess how risk varies in space and time and adapt their denning behaviour accordingly. We used the badger in Białowieża Forest, Poland, as a model for investigating how the denning behaviour of a fossorial mesocarnivore varies in response to short‐term large carnivore risk. To this end, we experimentally simulated perceived wolf presence outside 10 badger setts using audio playbacks of wolves (their howls). We assayed two behavioural measures of fear: badger emergence time from setts on the day playbacks were broadcast and their presence in setts on the day after. We found that neither badger emergence time nor next‐day sett use varied in response to wolf playbacks. The results of the present study contrast with a previous study of ours that found badgers used setts in areas with high landscape level perceived wolf risk less often than those in lower‐risk areas. Together, these papers' findings suggest that different spatiotemporal scales of perceived risk can have differential effects on badger behaviour. We conclude that rather than take risk avoidance measures at all risky times and places, badgers likely display a diversity of reactions to large carnivore presence that depend on the context and spatiotemporal scale of the risk being perceived.

## Graphical abstract

In this study, using a playback experiment, we investigated whether badger denning behaviour varies in response to the presence of wolves at their setts. Contrary to our hypotheses, we found that neither badger emergence time nor next‐day sett use varied with simulated wolf presence. These results contrast with the findings of a previous study of ours, which found that badgers used setts in areas with high landscape level perceived wolf risk less often than those in lower‐risk areas. Taken together, the results of these papers suggest that different spatiotemporal scales of wolf risk can have differential effects on badger behaviour.

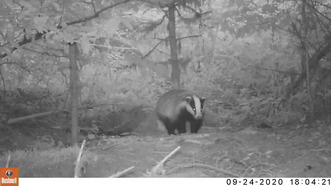

## 1 INTRODUCTION

Large, competitively dominant carnivores can suppress the abundance of sympatric mesocarnivores (Newsome et al., 2017; Prugh & Sivy, 2020). To reduce their chances of being killed, the latter can change their behaviour in response to spatial variation in perceived predation risk (Gaynor et al., 2019). One way they do this is by being fossorial, that is by hiding in burrows (Noonan et al., 2015). But in areas where large carnivores are present, fossorial mesocarnivores face a trade‐off between risk avoidance, food acquisition and reproduction (Lima & Dill, 1990; McArthur et al., 2014). They must arise overground to forage and mate while also minimising the likelihood of being killed by a predator through various antipredator behaviours. To maximise their fitness, it is imperative they assess how predation risk varies in space and time and adapt their denning behaviour accordingly.

In areas where they co‐occur with large carnivores, mesocarnivores navigate landscapes with dynamic spatiotemporal patterns of predation risk (Gaynor et al., 2019; Kohl et al., 2018; Palmer et al., 2022). To reduce their probability of being killed or disturbed, mesocarnivores can change a variety of behaviours, such as habitat use, temporal niches and vigilance (Gaynor et al., 2019) and they can do so at multiple spatiotemporal scales (Palmer et al., 2022). They can increase antipredator behaviour reactively during ‘risky times’, in response to short‐term variation in risk, that is when predators are in close proximity (‘risky‐times hypothesis’, Clinchy et al., 2016; Creel et al., 2008; Dröge et al., 2017). They can also adjust their behaviour proactively in risky places, in response to the long‐term risk associated with an area, that is where predators tend to kill prey or occur (‘risky‐places hypothesis’, Creel et al., 2008; Dröge et al., 2017; Nickel et al., 2020). Moreover, these aspects of risk can interact to produce patterns of mesocarnivore behavioural responses that are dynamic in space and time (Creel et al., 2008; Dröge et al., 2017; Palmer et al., 2022). Fossoriality is a common trait altering space use in mesocarnivores, but despite the obvious antipredator benefits it would seem to provide in helping mesocarnivores avoid predators, we still have a poor understanding of how denning behaviour varies in response to risk.

The European badger *Meles meles* is one of the most common fossorial mesocarnivores in Europe, and recent studies have used it as a model for investigating how mesocarnivores can vary their denning behaviour in the face of risk. We recently reported how the frequency badgers use setts varies in risky places, decreasing with increasing perceived landscape‐level wolf, *Canis lupus*, risk in Białowieża Forest (BF), Poland (Diserens et al., 2021). Clinchy et al. (2016) reported how badgers vary their denning behaviour during risky times, increasing their levels of antipredator behaviour at setts in the face of human but not wolf playbacks in Wytham Woods, UK. However, in areas lacking native large carnivores, such as Wytham Woods, mesocarnivores can become naïve to the cues of their predators, causing them to display fearless behaviour in the face of risk (Kuijper et al., 2016). With large mesocarnivore interactions being contingent on contextual factors such as predator presence and density (Haswell et al., 2017; Kuijper et al., 2016; McArthur et al., 2014), it remains unclear how badger denning behaviour varies during risky times in landscapes where they co‐occur with wolves.

Here, we investigated how the badger's denning behaviour varies with variation in short‐term perceived wolf risk (a risky time) in BF. In this forest, badgers have sympatrically occurred with wolves for centuries. Wolves hunt and kill badgers (Mysłajek et al., 2012; Olsson et al., 1997) and commandeer badger setts for their own pup‐rearing (Theuerkauf et al., 2003). Thus, we predicted that badgers fear the presence of wolves near their setts and hence modify their denning behaviour when wolves are perceived to be nearby. We simulated wolf presence near setts by carrying out a playback experiment exposing badgers to wolf sounds. Mesocarnivores can perceive predator presence using visual, olfactory and/or auditory cues (Hettena et al., 2014; Parsons et al., 2018; Vanak et al., 2009), and simulating risk using audio playbacks has proven to be a reliable and readily interpretable means of testing mesocarnivore responses to predator presence (Clinchy et al., 2016; Hettena et al., 2014; Suraci et al., 2016, 2019). We hypothesised that badgers would emerge from their setts later and use their setts less often after being exposed to the wolf audio playbacks.

## 2 MATERIALS AND METHODS

### 2.1 Study area

**FIGURE 1:**
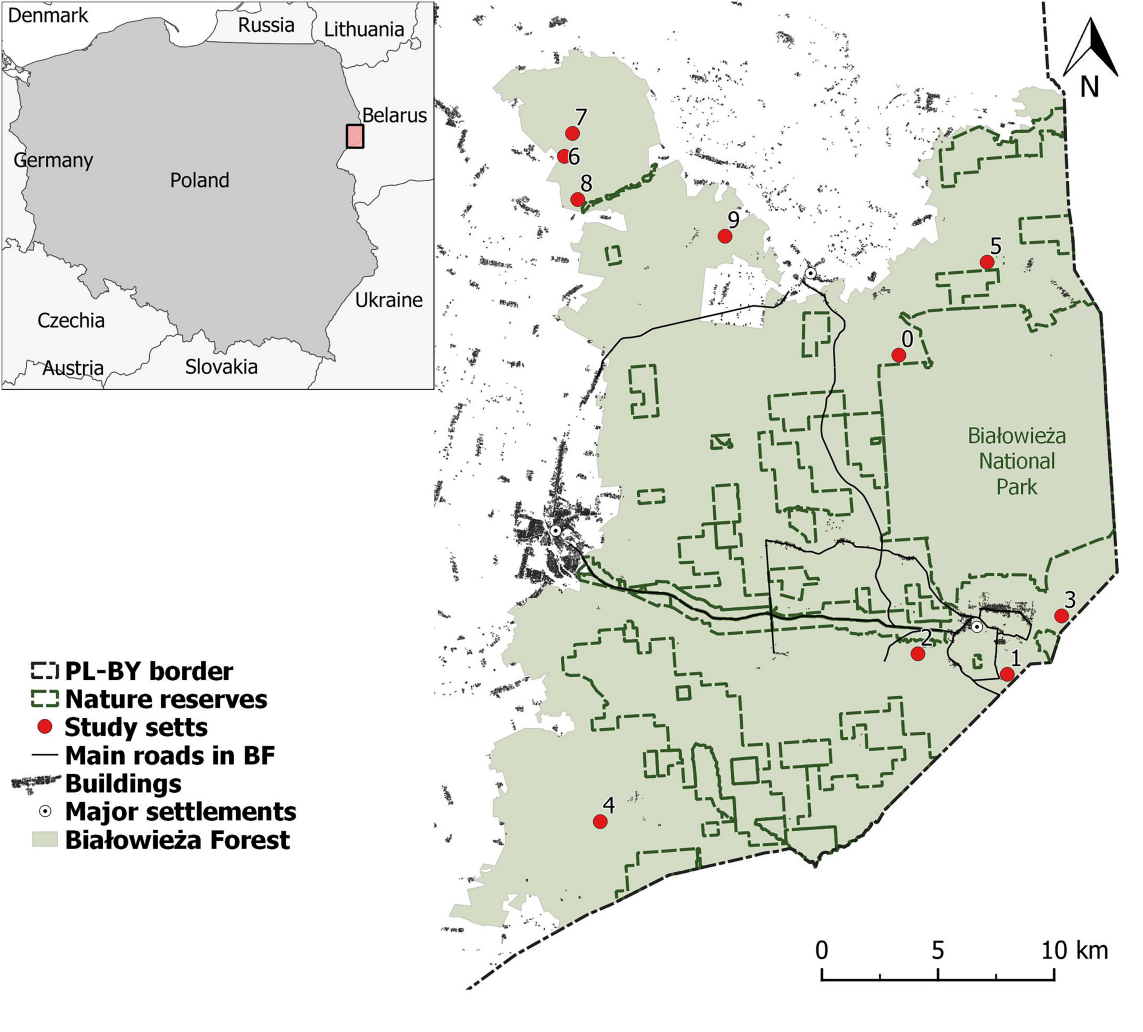
The location of the 10 setts monitored during the main part of the study. The numbering of the setts corresponds to the numbering in Tables S1 and S2. We generated the image in QGIS v 3.16.16 (www.qgis.org).

We carried out the study in BF, one of the best‐preserved forests in the European lowlands. The woodland spans the Polish–Belarusian border (52°30′–53° N, 23°30′–24°15′ E) and covers 1450 km^2^, with 600 km^2^ in Poland and 850 km^2^ in Belarus. The area is relatively flat (134–197 m a.s.l.), and its climate is transitional between Atlantic and continental types, with a mean annual temperature of 7°C and precipitation of 641 mm. Our study took place on the Polish side, whose forest habitats comprise oak–lime–hornbeam forest (56.5%), wet ash–alder forest (19.2%) and coniferous and mixed forest (17.9%), with open habitats covering the rest of the area (glades with meadows, riverside open sedge and reed marshes − 6.4%; calculated from Kwiatkowski, 1994). The best‐preserved tree stands are protected within Białowieża National Park, covering 18% (105 km^2^) of the Polish side, where hunting and forestry are banned. The rest is managed by the National Forest Holding ‘State Forests’ and subject to small‐scale clear cuts and hunting, but where also exists a network of nature reserves that protects well‐preserved old‐growth stands (22%, ca. 130 km^2^). A temporary ban on forestry activities meant that no logging occurred in BF over the field work period (ECJ, 2018). The studied badger setts were located across the Polish BF outside the national park and nature reserves (Figure 1). This area is freely accessible to hikers and cyclists and only open to cars with a permit, except for two public roads that intersect the forest.

### 2.2 Badger ecology and carnivore community structure in BF

The European badger (*Meles meles*) is a fossorial species that spends up to three quarters of its life in setts that are used for winter sleep, rest and reproduction (Kowalczyk et al., 2004; Kowalczyk, Jȩdrzejewska, & Zalewski, 2003). In BF, it occurs at densities of 1.52–2.11 badgers per 10 km^2^ (Kowalczyk, Zalewski, et al., 2003), and each social group uses an average of 9 different setts and shelters (e.g., hollow trees) per territory, one of which is a ‘main sett’ that is used most frequently on an annual basis (Kowalczyk et al., 2004). Badger denning behaviour is plastic, and individuals can, *inter‐alia*, modify their choice of sett for daytime rest and the time they emerge from a sett in the evening (Kowalczyk, Jȩdrzejewska, & Zalewski, 2003). In BF, the badger occurs within a carnivore community of 11 species (Jędrzejewska & Jędrzejewski, 1998), and it coexists with two large carnivores, the wolf and lynx *Lynx lynx*, which have been strictly protected and not hunted on the Polish side of BF since the 1990s. Three to four wolf packs each with 5–12 individuals occur on the Polish side of BF (Bubnicki et al., 2019; Jędrzejewski et al., 2007). The home ranges of wolves and lynx cover the entire forest complex (Jędrzejewski et al., 2007; Schmidt et al., 2009), but they show clear gradients in space use; hence, the probability of encountering them varies across the landscape (Bubnicki et al., 2019). Space use by wolves and lynx overlaps to a large degree (Bubnicki et al., 2019), and they do not spatially avoid each other (Schmidt et al., 2009). A recent study found that the lynx occurs at much lower densities than the wolf (Bubnicki et al., 2019). The lynx could potentially pose a threat to badgers, but we are unaware of any evidence showing they disturb or kill badgers in this system. A brown bear, *Ursus arctos*, was documented in the forest shortly before the field study period (Diserens et al., 2020). However, there have been no sightings of females with cubs, indicating the species has no permanent presence in the forest. In BF, aside from being subject to top‐down pressure from wolves, badgers have historically been subject to hunting and poaching, and their setts subject to being dug up by humans (Jędrzejewska & Jędrzejewski, 1998; Kowalczyk et al., 2000). Nowadays, badgers are rarely hunted, but poaching likely occasionally occurs, and they are a game species in other parts of Poland.

### 2.3 Selection of badger setts

We ran a pilot survey to identify setts that were in active use. We knew the locations of 67 badger setts (including main, secondary and occasional sets) in BF from previous studies (Kowalczyk, Zalewski, et al., 2003; Obidziński et al., 2013). We selected 16 of them on the basis that they (i) were outside the national park and nature reserves (as we were not permitted to work inside them) and (ii) had evidence of recent activity (paths, signs of digging or tracks). We deployed one camera trap (LTL Acorn SGN‐5310 M) at each sett 5–15 m away from the entrance that looked the most used. The cameras were programmed to take a 60‐s video when triggered by motion (as detected via changes in temperature). Setts were monitored for 7–10 days between 11th and 20th August 2020. Of the 16 setts studied during the pilot study, we recorded badgers at 10, which formed the basis of the main field study.

### 2.4 Simulating wolf presence and playback field experiment

The experimental set‐up was initially aimed at testing whether badger denning behaviour varies with (1) wolf playbacks on a given day (to test the badgers' immediate response to perceived wolf presence) and (2) two intensities of playbacks (low and high intensity) broadcast over 6 days (to test the badgers' response to varying levels of longer‐term perceived wolf presence). We failed to collect sufficient data to test aim two; however, in this section we describe how we aimed to investigate it, as otherwise the field study set up would not make sense to the reader. We subjected the 10 setts to four consecutive 6‐day long sessions, with one of four treatments being applied during each: high‐intensity wolf, low‐intensity wolf, high‐intensity tawny owl (*Strix aluco*) and low‐intensity tawny owl (the latter two treatments being controls). During the high‐intensity treatments, playbacks were broadcast every day during a session, whereas during the low‐intensity treatments, only on two evenings per session, on the 1st and 4th days. Under the high‐intensity treatment, we aimed to simulate regular, repeated visits of wolves to the vicinity of setts over multiple days; it is natural for wolves to revisit an area when it becomes, for example, their rendezvous site (Theuerkauf et al., 2003), which is also where they howl most often (Nowak et al., 2007). Under the low‐intensity treatment, we aimed to simulate transient wolf presence.

The playbacks were broadcast from 1 to 0.5 h before sunset and lasted for 4 h. We chose this start time as at the end of summer badgers emerge from their setts around sunset (Kowalczyk, Jȩdrzejewska, & Zalewski, 2003), which also coincides with the natural evening increase in wolf howling activity (Nowak et al., 2007). We used multiple exemplars of each playback type, that is different sample sounds of the wolf or control species: 9 wolf exemplars and 10 control exemplars. We used multiple exemplars so as to induce responses to a ‘class’ of sounds rather than the specifics of any particular sound. For the wolf treatment, we used 9 of the 10 wolf exemplars used in Clinchy et al. (2016). The wolf exemplars comprised a variety of howls, both of lone individuals and packs howling together, between 18 and 78 s long. As the control, we used calls of the tawny owl, a sympatric predatory bird, as a non‐threatening negative control that does not predate badgers. Badgers should have been familiar with the sound, as this bird occurs commonly throughout the forest (Jędrzejewska & Jędrzejewski, 1998). The tawny owl exemplars originated from the website Xeno‐Canto (www.xeno‐canto.org) and comprised a variety of 10‐s long records of the species' calls.

The sound files were arranged in Audacity v 2.4.2 (Audacity Team, 2020). We created 12 4‐h‐long tracks, six per type of species (wolf or tawny owl). Tracks of a given type differed in their composition of exemplars. Tracks were subdivided into eight half‐hour segments. At a random timepoint within each segment, a randomly chosen exemplar of the relevant species was encoded. We chose this regularity of howling to indicate the persistent presence of wolves in the vicinity of the wolf sett on a given evening, and this howl frequency (8 howls per 24 h) is within the natural range of wolf call rates (e.g. 10 howls per 24 h for wolves in YNP; McIntyre et al., 2017). The files were normalised to match peak amplitudes amongst treatments using the normalise function in Audacity to ensure the reactions to our treatments were unrelated to variability in sound intensity between or within treatments. The same tracks were used for both the high‐ and low‐intensity treatments (i.e., in both intensities, the howl frequency was the same on days playbacks were programmed to be played, which was 2 days per week for the low‐intensity treatment and 6 days per week for the high‐intensity treatment). On days playbacks were tabled to be broadcast, one of the tracks of the appropriate type was randomly chosen to be broadcast at each sett. In total, over the field study period, each sett was subject to wolf playbacks on 16 days, control playbacks on 16 days and no playbacks on 8 days.

We carried out the field study in September 2020. The setts were subject to treatments in a random order, except that two of the same species of treatments could not follow each other (e.g. a session subject to a high‐intensity wolf treatment could not follow one subject to a low‐intensity wolf treatment and vice versa). The playbacks were broadcast from water‐resistant Mp3 speakers (model: StormMp3, brand: ToiletTree products) at a distance of <50 m from active entrances. The speakers broadcast at a peak volume of 70 dB at 1 m, as measured on a smart phone using the Android application Sound Meter (developer: Abc Apps). Sounds were not broadcast from the same location on any two consecutive days. Speakers only held charge for 72 h on a fresh set of batteries, so we visited plots every 3 days between 09:00 and an hour before sunset. At each sett, we placed two speakers, so that sounds were broadcast from one location on the first and third days, but from a second location on the second day. On the fourth day, the speakers were moved 5–15 metres and the batteries replaced. The sounds were then broadcast from a third location on the fourth and sixth days and from a fourth location on the fifth day. At each badger sett, we deployed 2–6 motion‐activated camera traps (LTL Acorn SGN‐5310 M and Bushnell Trophy Cam HD 2013) on trees 5–10 m away from entrances. We mounted varying numbers of camera traps, as setts can cover a wide area and have varying numbers of entrances not all of which are used continually. We tried to find all active entrances, so that each of them was in view of a camera. The cameras were set to record 30‐s videos at 0‐s intervals upon being triggered by motion.

### 2.5 Analysis of badger behavioural responses to wolf playbacks

The camera trap videos were uploaded to Trapper (www.os‐conservation.org), a web‐based application for managing camera trap data (Bubnicki et al., 2019). We used Trapper to classify the videos, recording the identity of species and the date and time animals were active. A single person classified the videos. The measures of denning behaviour assayed were the time of day that badgers first emerged from their sett after a given playback was played (emergence time hereinafter) and the presence/absence of badgers in setts on the day after (next‐day sett use hereinafter). These measures were chosen because previous studies have shown badger emergence time can vary in response to human playbacks (Clinchy et al., 2016) or disturbance (Lindsay & Macdonald, 1985; Tuyttens et al., 2001), and a previous study of ours showed that sett use can vary with perceived landscape‐level wolf risk (Diserens et al., 2021). To extract the presence and absence of badgers on each study day at each sett from the camera trap dataset, we built an occupancy matrix using camtrapR (Niedballa et al., 2016). A day was considered to be from 00:00 on 1 day until 00:00 on the next. To determine the time at which badgers began their activity for the day, we manually inspected the data to extract the time at which the first badger was recorded on each day at each sett. We then calculated the emergence time as the time difference between the time of the first activity and the time playbacks started playing (emergence time = time of first activity – (sunset time – 30 min)). We had to remove several inadequate records from the emergence time, and next‐day sett use datasets. We excluded from both datasets records when badgers emerged earlier than 30 min before sunset (*N* = 4), as these represented days when badgers emerged before playbacks were broadcast. We also excluded from both datasets two outlying records — these videos were recorded 2–3 h after the average emergence time, and we assumed this was caused by the camera traps failing to record the initial emergence of badgers earlier in the evening or because badgers had come from elsewhere, having spent the day resting at a different location. We excluded from only the emergence time dataset 1 record where badgers swiftly returned to their setts after becoming active; in this case, there were no further records to note the actual emergence time. As in most cases the badgers simply emerged from their setts and walked off, we were unable to carry out detailed behavioural analysis (e.g., of vigilance levels).

Badgers were present in setts at radically varying frequencies, being recorded at some setts on only 0, 1 or 2 days per playback type (Figure S1). To analyse the immediate response to the playbacks, we noted the type of playback (wolf, tawny owl, or no playback) that was broadcast on the days badgers were present in setts — which we refer to as playback type hereinafter. We obtained from 0 to 8 emergence times and sett use records for each playback type at each sett (Table S1). To prevent including pseudoreplicated datapoints, we averaged the records for each playback type at each sett, so that each sett had one emergence time and next‐day sett use value for days when wolf, tawny owl and no playbacks were broadcast. We carried out non‐parametric Kruskal–Wallis tests to compare average emergence times and next‐day sett use between playback types at each sett. Statistics were carried out using the kruskal test function in R v 4.0.3 (R Core Team, 2021).

We additionally attempted to carry out mixed‐effects modelling to explain both response variables. We ran a linear mixed‐effect model on the emergence time data and a generalised binomial mixed‐effects model on the next‐day sett use data. In both models, we included sett ID as a random factor to account for sett level differences caused by factors such as vegetation structure, distance to settlements and landscape‐level wolf risk (Bubnicki et al., 2019), amongst other factors. We also included the day of the year in the emergence time model, as this response variable is known to vary with time of year (Kowalczyk, Jȩdrzejewska, & Zalewski, 2003). However, carrying out diagnostics using the R package DHARMa v 0.4.6 (Hartig, 2022) showed that these models fit rather poorly to the data. For our readers' interest we have included in the supplementary material the results of these models (Tables S3 and S4) and their diagnostic plots (Figures S2 and S3). Statistics were carried out in R packages lme4 v 1.1‐29 (Bates et al., 2015; emergence time model) and GlmmTMB v 1.1.3 (Brooks et al., 2017; next‐day sett use model).

## 3 RESULTS

We recorded 516 videos of badgers, and we noted them emerging from setts on 63 sett days. On these evenings, we knew badgers were in their setts during the time the playbacks were being broadcast, so these datapoints formed the basis of the next day sett use and emergence time analyses. After removing inadequate results, we obtained values for emergence time on 56 days and next day sett use on 57 days. After averaging at the level of the playback type at each sett, we obtained emergence times and next day sett use values for 5 (wolf playbacks), 6 (tawny owl) and 7 setts (no playback) (Table S2).

**FIGURE 2:**
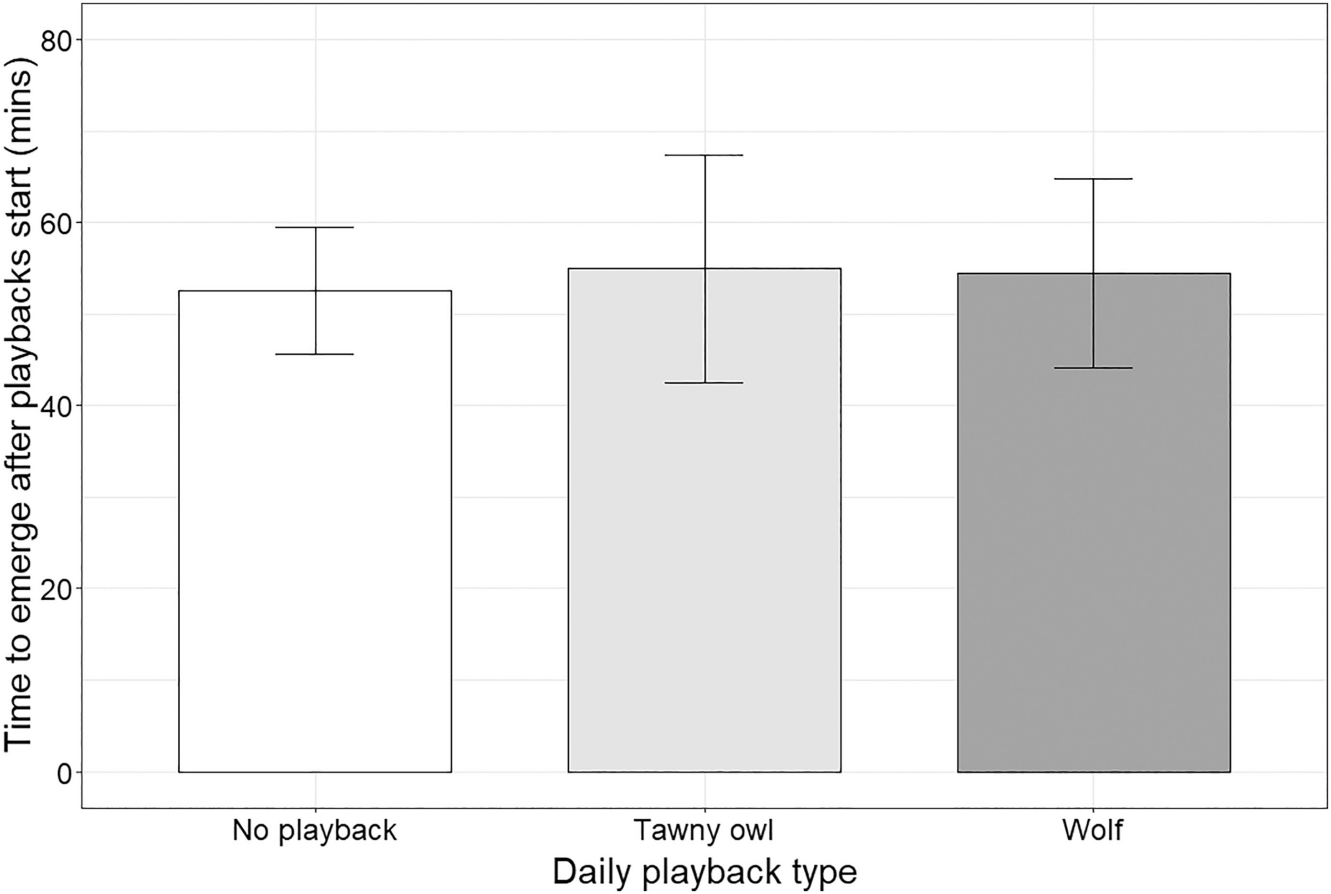
Effect of playback type on the time badgers emerged from setts. Values are means ± SE.

**FIGURE 3:**
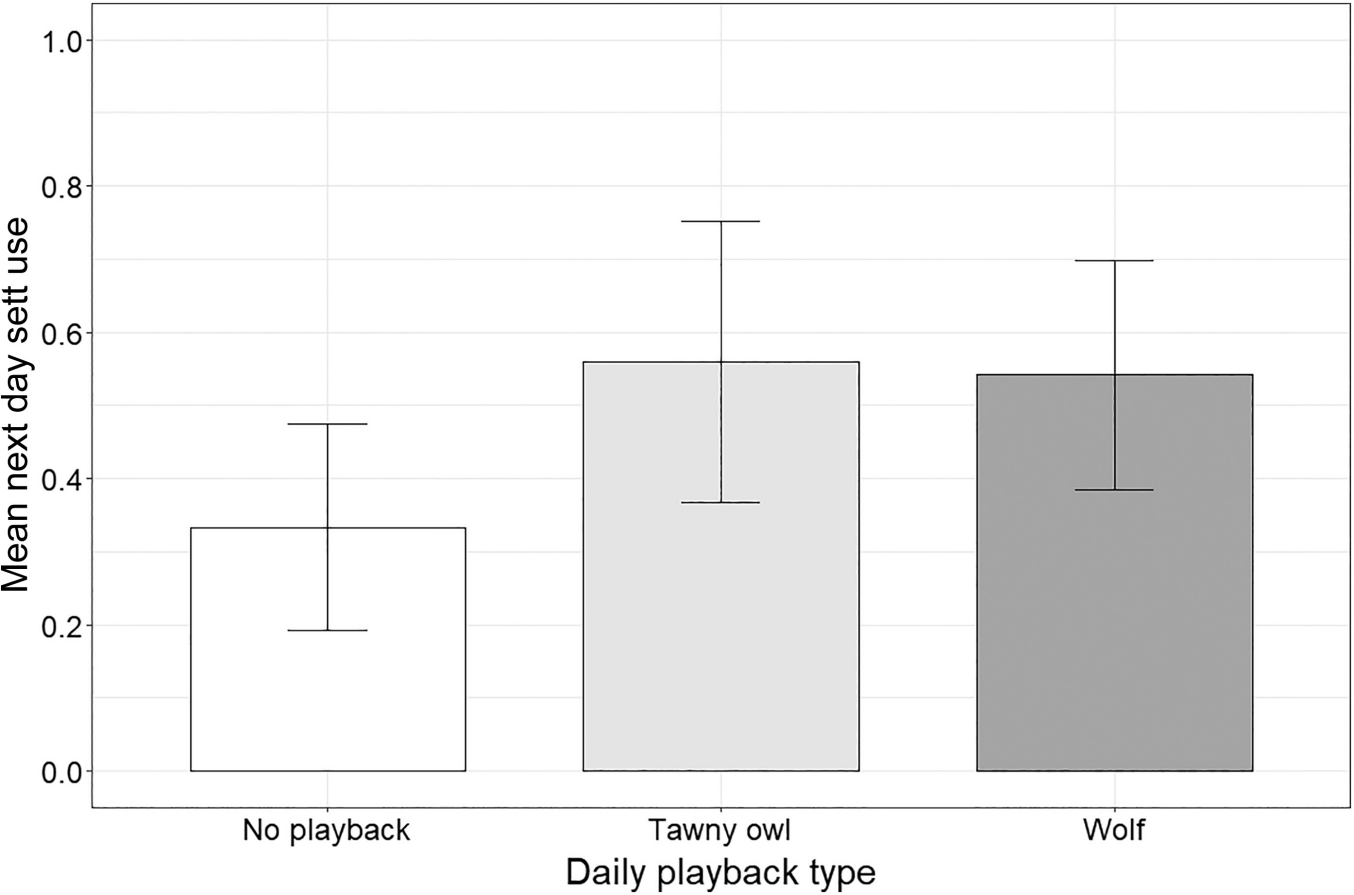
Effect of playback type on the presence of badgers in setts on the day after playbacks of a given type were broadcast. Values are means ± SE.

Emergence time did not vary between playback types (Kruskal–Wallis *χ* ^2^ = 0.100, df = 2, *p* = .951, Figure 2). Next day sett use also did not vary between playback types (Kruskal–Wallis *χ* ^2^ = 1.2412, df = 2, *p*‐value = .538, Figure 3).

## 4 DISCUSSION

We tested whether badgers vary their denning behaviour in response to the simulated presence of wolves (a risky time) near their setts. Hearing the sounds of wolves howling affected neither of the two titrated measures of fear, suggesting that the perceived short‐term presence of wolves in the vicinity of setts is not an important driver of badger‐denning behaviour in this system. By contrast, in our previous study (Diserens et al., 2021), we found that badger sett use (over a 2 month period) decreased with increasing landscape‐level wolf encounter rates (i.e., it decreased in risky places). Together, the results of these studies suggest that risky times and places have differential effects on badger behaviour: rather than simply fearing and avoiding times and places of high perceived risk, badgers seem to display a diversity of reactions to different spatiotemporal scales of predator presence, ranging from avoidance to tolerance. Below we discuss why badgers appear to tolerate the perceived short‐term presence of wolves near their setts.

If badger denning behaviour varied with perceived wolf presence, we expected badgers to delay their emergence from setts after hearing wolf playbacks and avoid using that sett the day after. However, in contrast to recent playback studies that found mesocarnivores display antipredator behaviour after hearing playbacks of their predators (Clinchy et al., 2016; Suraci et al., 2016; Suraci, Roberts, et al., 2017), neither emergence time nor next‐day sett use varied in response to wolf playbacks. To reduce risk, it is possible that badgers used some other risk avoidance behavioural response that we could not observe in this study. They could, for example, have emerged overground and foraged in close proximity to the safety of their setts (but outside the range of our camera traps) or have increased their vigilance levels for a period of time after emerging (Blanchard et al., 2018). Nevertheless, that they emerged from their setts in the face of wolf playbacks and returned to the same setts the next day suggests that they perceive a wolf present in the vicinity of their setts to pose a low level of risk. It also suggests that in BF, badgers perceive the costs of missing foraging opportunities to be higher than the benefits of mitigating this risk by sheltering safely in setts. In our previous study, we found badger sett use (over a 2 month period) decreased with increasing landscape‐level wolf encounter rates, a long‐term perceived risk factor that has been stable in BF for decades (Bubnicki et al., 2019). This contrasting response of badgers to different scales of perceived risk suggests that badgers in BF respond to short‐term (a risky time) and long‐term wolf presence (risky places) in different ways, as has been found in other species (Diserens et al., 2022; Dröge et al., 2017; Palmer et al., 2022).

Our results both compare and contrast with those of Clinchy et al. (2016), the only other playback study carried out near a burrow of any species. Similarly, their study also found that badgers failed to vary their denning behaviour (emergence time or vigilance) after hearing wolf playbacks. The authors hypothesised that the lack of response to wolf playbacks resulted from the badger's naivety towards wolves, which were extirpated 400 years ago (Clinchy et al., 2016). It is therefore striking that badgers in both BF and Wytham Woods do not vary their denning behaviour in response to wolf playbacks, the former being an area where the badger has co‐occurred with the wolf for centuries (Jędrzejewska & Jędrzejewski, 1998). The similarity of the results in both of these study areas suggests that the badger's lack of response to perceived wolf presence in the vicinity of setts may be a more universal phenomenon and not just due to naivety. By contrast, however, Clinchy et al. (2016) found that badgers were frightened after hearing human playbacks, not emerging from their setts till after their broadcasting had ended (over 2 h). We found that badgers remain fearless in the face of wolf playbacks in BF, suggesting that the perceived costs associated with wolf presence in BF are far lower than the costs associated with human presence in Wytham Woods, which seems to support Clinchy et al.'s (2016) thesis that humans are far more frightening to badgers than wolves. Although the apparent lack of fear badgers displayed in response wolf playbacks in BF could also be caused by the much lower abundances of badger food in BF, making the costs of hiding in setts for prolonged periods much higher than in Wytham Woods, where earthworm sizes and abundances ha^−1^ are several fold higher than in BF (Hofer, 1988; Kowalczyk, Zalewski, et al., 2003). We should also point out that Clinchy et al. (2016) used a different frequency of playbacks than we did — they broadcast predator playbacks c. every 2 min for 2 h compared with once every half hour for 4 h in our study. Thus, it is also possible that the badgers' fearfulness of humans in their study was due to their having simulated a much higher intensity level of perceived risk.

It is surprising that badgers appeared to perceive wolf presence as representing a low level of risk, as wolves hunt and kill badgers (Mysłajek et al., 2012; Olsson et al., 1997). However, in late summer in BF, wolves may not pose much of a threat to badgers, which in turn, have little need to be risk averse. At this time of year, wolves may have little motivation to kill badgers, as competition between badgers and wolves is likely to be low: badgers do not hunt the same animals as wolves (Jędrzejewska & Jędrzejewski, 1998), do not often scavenge on wolf kills (badgers scavenge on only ca. 5% of carcasses in BF, Selva, 2004), nor do they compete for den sites outside of the wolf denning season (May–July; Jędrzejewski et al., 2001). Moreover, ungulates occur at relatively high densities throughout the forest (Bubnicki et al., 2019); thus, the badger likely suffers limited predation from wolves. Indeed, badgers occur rarely in the wolf diet in BF (Jędrzejewska & Jędrzejewski, 1998). By contrast, ungulates have been found to perceive fear in response to wolf howls (Crawford et al., 2022; Hettena et al., 2014; Widén et al., 2022). However, ungulates form the majority of the wolf diet in many areas (e.g. they form c. 97% of the biomass in the wolf diet in BF; Jędrzejewska & Jędrzejewski, 1998) and thus have a greater incentive to fear and evade wolves. Still, it is unlikely badgers remain fearless in the face of a wolf's presence in all situations. Badgers may be more fearful when they are away from the safety of their setts, for example when foraging. Their fear threshold for taking evasive measures may also require them to perceive a combination of wolf cues, for example visual and/or odour or simply the meeting of a real wolf (Scheinin et al., 2006; Vanak et al., 2009). We make some recommendations for studying the effects of different scales of perceived risk in other contexts on badger behaviour below.

It could be argued that the speaker‐playback system used in our study did not accurately simulate wolf presence or their howling; however, several studies have found that wolf howls experimentally broadcast from store‐purchased speakers inspire fear in wolf prey (Crawford et al., 2022; Hettena et al., 2014; Widén et al., 2022), and thus we regard the results of the present study as reliable indicators of badger responses to perceived wolf presence. We originally aimed to also test whether repeated wolf playbacks over 6 days (prolonged perceived wolf presence in the vicinity of setts) shapes badger denning behaviour; however, we did not collect enough data to test this. The small amount of data collected in our study was due to our methodology being inspired by that used in Clinchy et al. (2016). However, in their study area, Wytham Woods, badgers have a different ecology, whereby they use only one sett for daytime rest and therefore have a more predictable behaviour that allows data to be collected each study day at each sett. The differences in badger spatial organisation across Europe, such as those between BF and Wytham Woods, have been suggested to be a consequence of variation in food availability between study areas (Kowalczyk, Zalewski, et al., 2003). Yet the presence and densities of large carnivores also vary across the continent, and the role of risk in determining these differences in badger ecology has not been examined, making this an interesting potential topic for future studies. It is possible that the badgers' response to wolf presence varies depending on where along the perceived landscape‐level wolf risk gradient a sett lies (Bubnicki et al., 2019; Diserens et al., 2022; Lima & Bednekoff, 1999). (Bubnicki et al., 2019; Diserens et al., 2021); however, the 5 setts for which we obtained responses to wolf playbacks were too poorly distributed across the landscape‐level risk gradient (Figure S4) to test for any context‐dependent effect. To further test the effects of different spatiotemporal scales of large carnivore risk on badger behaviour, future studies should consider exploring badger behavioural responses to the following: (1) combinations of predator cues or even direct predator presence; (2) large carnivore risk in a variety of contexts, for example away from setts at foraging patches; and (3) short‐term risk at a variety of points along a landscape level perceived risk gradient, to test whether risky times and risky places interact to shape badger behaviour. Such studies could be done with telemetry (GPS collaring both large carnivores and badgers simultaneously) or using a motion‐activated speaker‐sound system (Suraci, Clinchy, et al., 2017) deployed along forest roads or at artificial feeding sites.

The large carnivore recolonisation of the European lowlands has the potential to restore ecological functionality via the creation of a landscape of fear that suppresses the behaviour of mesocarnivores (Kuijper et al., 2016; Ritchie & Johnson, 2009), amongst other effects. But recent studies have shown that when perceiving risk inspired by large carnivores, mesocarnivores can show contrasting behavioural responses, varying from risk averse to risk tolerant. While several playback experiments have found mesocarnivores increase their antipredator activity after hearing predator cues (Clinchy et al., 2016; Suraci et al., 2016; Suraci, Roberts, et al., 2017). Other works have found mesocarnivores to tolerate (Diserens et al., 2022) or to even be attracted to the presence or cues of their predators (Ruprecht et al., 2021; Sivy et al., 2017). Our studies (the present study and Diserens et al., 2021) fit into this mixed picture, as we have observed badgers responding with both risk‐averse and risk‐tolerant denning behaviour to different spatiotemporal scales of perceived risk in BF. That badgers remain unreactive during risky times (the results of the present study) but not in risky places (Diserens et al., 2021) highlights the importance of testing the responses of prey and mesocarnivores to perceived risk at multiple scales (Palmer et al., 2022). We conclude that rather than take risk avoidance measures at all risky times and places, badgers are likely to display a diversity of reactions to large carnivore presence that depends on the context (e.g., the earlier mentioned seasonal factors) (Haswell et al., 2017; Kuijper et al., 2016) and spatiotemporal scale of the risk being perceived (Palmer et al., 2022).

## AUTHOR CONTRIBUTIONS

**Tom A. Diserens:** Conceptualization (equal); data curation (lead); formal analysis (lead); investigation (lead); methodology (equal); project administration (lead); visualization (lead); writing – original draft (lead); writing – review and editing (lead). **Marcin Churski:** Conceptualization (equal); formal analysis (supporting); funding acquisition (supporting); methodology (equal); supervision (supporting); writing – review and editing (supporting). **Jakub W. Bubnicki:** Conceptualization (supporting); formal analysis (supporting); methodology (supporting); resources (equal); software (equal). **Andrzej Zalewski:** Conceptualization (supporting); methodology (supporting); writing – review and editing (supporting). **Marcin Brzeziński:** Conceptualization (supporting); methodology (supporting); writing – review and editing (supporting). **Dries P. J. Kuijper:** Conceptualization (equal); formal analysis (supporting); funding acquisition (lead); methodology (equal); resources (equal); supervision (lead); writing – original draft (supporting); writing – review and editing (supporting).

## CONFLICT OF INTEREST STATEMENT

The authors declare no competing interests.

## Supporting information


Figure S1

Figure S2

Figure S3

Figure S4

Table S1

## Data Availability

The datasets used and/or analysed in the present study are available in Table S1.
