# Supplementary figures and images for: Badgers remain fearless in the face of simulated wolf presence near their setts
Source: Ecol Evol. 2024 Jan 4;14(1):e10654. doi: 10.1002/ece3.10654 (PMC10767146; doi:10.1002/ece3.10654)

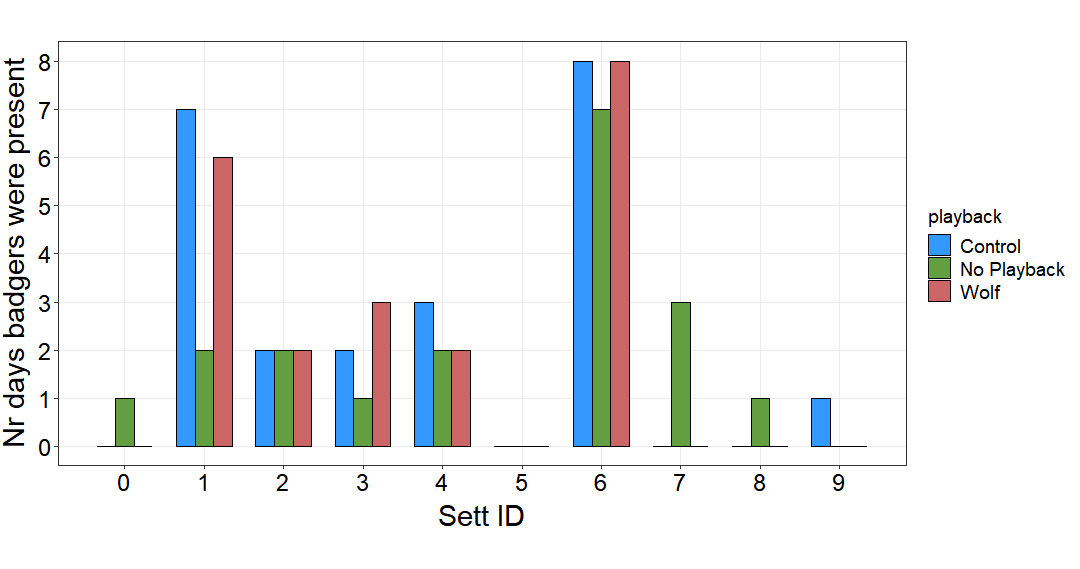

Supplement: Supplementary file 1 — Figure S1 [file ECE3-14-e10654-s003.png]

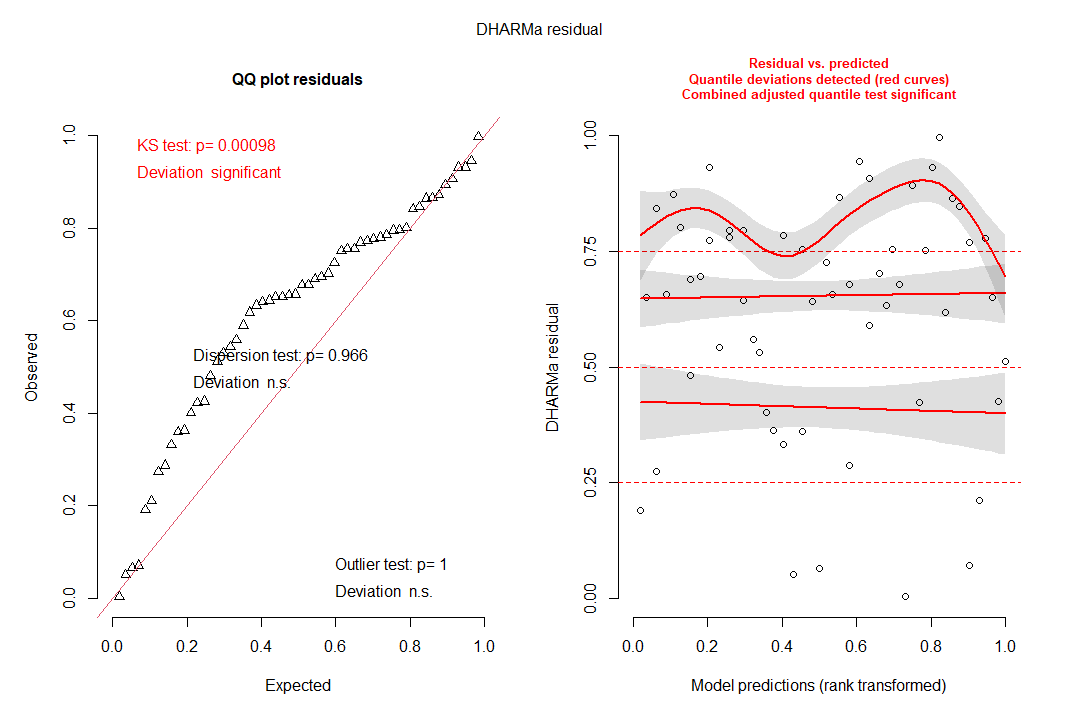

Supplement: Supplementary file 2 — Figure S2 [file ECE3-14-e10654-s005.png]

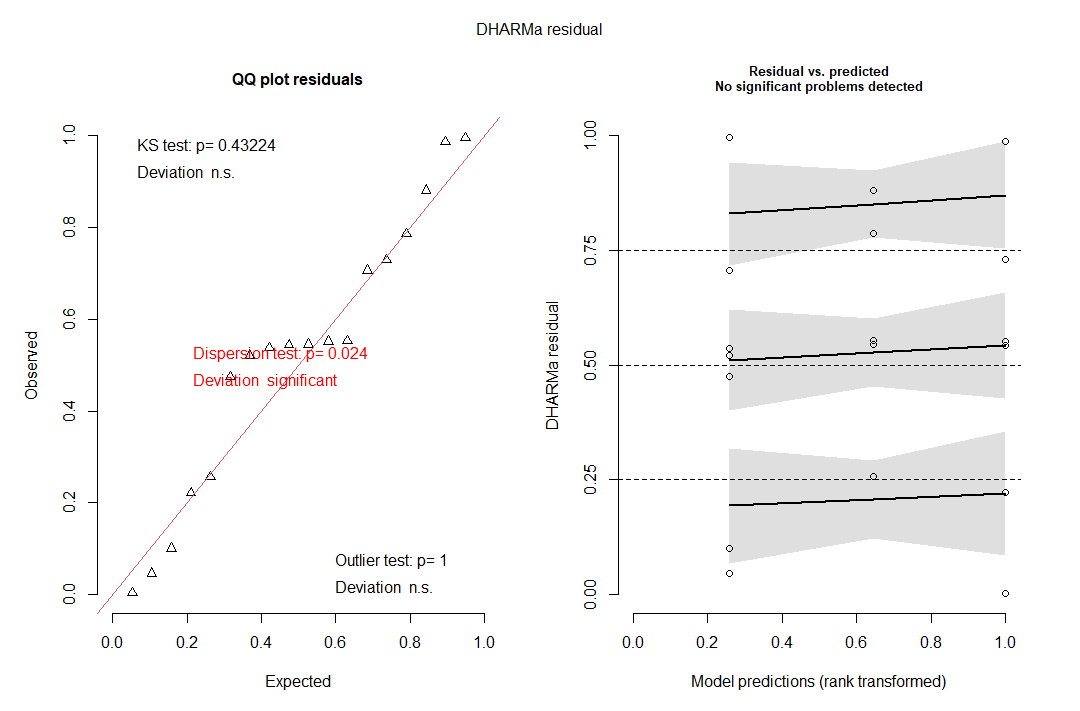

Supplement: Supplementary file 3 — Figure S3 [file ECE3-14-e10654-s001.png]

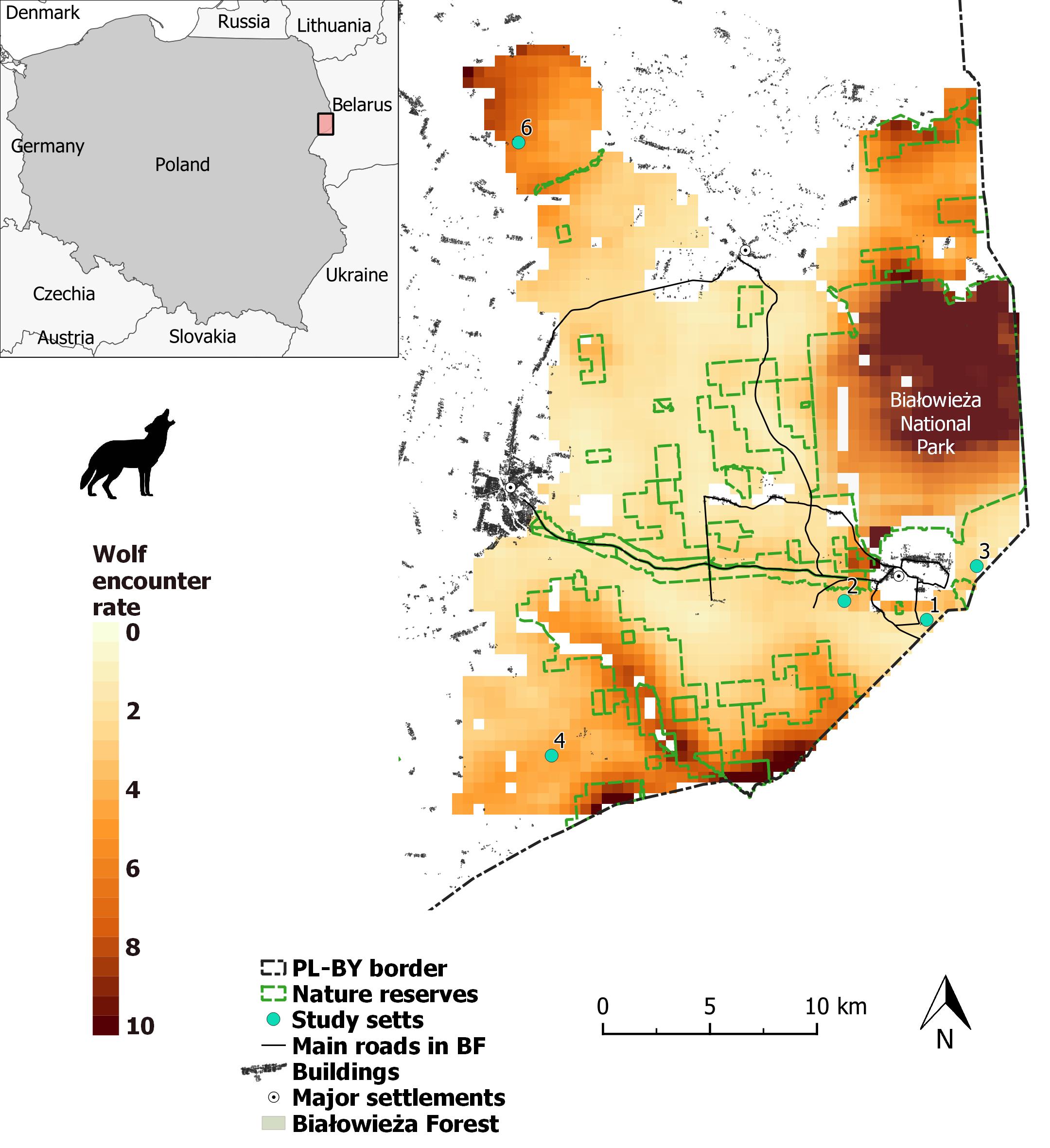

Supplement: Supplementary file 4 — Figure S4 [file ECE3-14-e10654-s004.jpeg]
