# Supplementary material for: Badgers remain fearless in the face of simulated wolf presence near their setts
Source: Ecol Evol. 2024 Jan 4;14(1):e10654. doi: 10.1002/ece3.10654 (PMC10767146; doi:10.1002/ece3.10654)
Supplement: Supplementary file 5 — Table S1 [file ECE3-14-e10654-s002.docx]

**Appendices**

**Table S1** Raw data for emergence time and next day sett use response variables. Treatment refers to the treatment applied during the session the datapoint was collected. Playback type refers to the type of track played on the day the datapoint was gathered (wolf, control, no playback). Next day sett use refers to the presence of badgers on the day after the badgers heard the playbacks broadcast outside their setts (1 = present, 0 = absent). Record date and time refer to the date and time the badgers emerged from their setts on a given day. Playback start time is sunset time -30 min, which is the time playbacks started on given days. Emergence time is the time it took for badgers to emerge from setts after the playbacks started. Unreliable refers to datapoints omitted from the final dataset and analysis, with the comment column explaining why it was omitted.

| Sett # | Treatment | Playback type | Record date | Sunset | Playback start time | Record time | Emergence time | Next day sett use | Unreliable | Comment |
| --- | --- | --- | --- | --- | --- | --- | --- | --- | --- | --- |
| 1 | High control | Control | 07/09/2020 | 19:00 | 18:30 | 19:29 | 00:59 | 1 | No | NA |
| 1 | High control | Control | 08/09/2020 | 18:58 | 18:28 | 19:17 | 00:49 | 1 | No | NA |
| 1 | High control | Control | 09/09/2020 | 18:56 | 18:26 | 19:33 | 01:07 | 1 | No | NA |
| 1 | High control | Control | 10/09/2020 | 18:53 | 18:23 | 19:13 | 00:50 | 1 | No | NA |
| 1 | High control | Control | 11/09/2020 | 18:51 | 18:21 | 19:04 | 00:43 | 1 | No | NA |
| 1 | High control | Control | 12/09/2020 | 18:49 | 18:19 | 19:02 | 00:43 | 1 | No | NA |
| 1 | Low wolf | Wolf | 13/09/2020 | 18:46 | 18:16 | 19:00 | 00:44 | 1 | No | NA |
| 1 | Low wolf | No Playback | 14/09/2020 | 18:44 | 18:14 | 18:44 | 00:30 | 0 | No | NA |
| 1 | Low wolf | Wolf | 16/09/2020 | 18:39 | 18:09 | 19:13 | 01:04 | 0 | No | NA |
| 1 | Low control | Control | 19/09/2020 | 18:32 | 18:02 | 19:03 | 01:01 | 0 | No | NA |
| 1 | Low control | No playback | 21/09/2020 | 18:27 | 17:57 | 18:49 | 00:52 | 1 | No | NA |
| 1 | High wolf | Wolf | 27/09/2020 | 18:12 | 17:42 | 18:11 | 00:29 | 1 | No | NA |
| 1 | High wolf | Wolf | 28/09/2020 | 18:10 | 17:40 | 19:00 | 01:20 | 1 | No | NA |
| 1 | High wolf | Wolf | 29/09/2020 | 18:08 | 17:38 | 18:39 | 01:01 | 1 | No | NA |
| 1 | High wolf | Wolf | 30/09/2020 | 18:05 | 17:35 | 18:40 | 01:05 | 1 | No | NA |
| 2 | High wolf | Wolf | 02/09/2020 | 19:12 | 18:42 | 19:53 | 01:11 | 1 | No | NA |
| 2 | High wolf | Wolf | 07/09/2020 | 19:00 | 18:30 | 18:59 | 00:29 | 0 | Yes | Emerged too early |
| 2 | Low control | Control | 09/09/2020 | 18:56 | 18:26 | 19:22 | 00:56 | 0 | No | NA |
| 2 | Low wolf | No playback | 19/09/2020 | 18:32 | 18:02 | 19:24 | 01:22 | 1 | No | NA |
| 2 | Low wolf | No playback | 20/09/2020 | 18:29 | 17:59 | 19:05 | 01:06 | 0 | No | NA |
| 2 | High control | Control | 25/09/2020 | 18:17 | 17:47 | 18:44 | 00:57 | 1 | No | NA |
| 3 | High wolf | Wolf | 07/09/2020 | 19:00 | 18:30 | 20:51 | 02:21 | 0 | Yes | Outlier |
| 3 | High wolf | Wolf | 10/09/2020 | 18:53 | 18:23 | 19:03 | 00:40 | 1 | No | NA |
| 3 | High wolf | Wolf | 11/09/2020 | 18:51 | 18:21 | 18:37 | 00:16 | 0 | No | NA |
| 3 | Low control | No playback | 17/09/2020 | 18:37 | 18:07 | 18:52 | 00:45 | 0 | No | NA |
| 3 | High control | Control | 26/09/2020 | 18:15 | 17:45 | 19:19 | 01:34 | 1 | No | NA |
| 3 | High control | Control | 27/09/2020 | 18:12 | 17:42 | 21:59 | 04:17 | 0 | Yes | Outlier |
| 4 | Low control | No playback | 11/09/2020 | 18:51 | 18:21 | 18:07 | NA | 0 | Yes | Emerged too early |
| 4 | High wolf | Wolf | 14/09/2020 | 18:44 | 18:14 | 18:36 | 00:22 | 0 | No | NA |
| 4 | High wolf | Wolf | 16/09/2020 | 18:39 | 18:09 | 18:52 | 00:43 | 0 | No | NA |
| 4 | High control | Control | 19/09/2020 | 18:32 | 18:02 | 18:00 | NA | 0 | Yes | Emerged too early |
| 4 | High control | Control | 21/09/2020 | 18:27 | 17:57 | 17:45 | NA | 0 | Yes | Emerged too early |
| 4 | High control | Control | 24/09/2020 | 18:20 | 17:50 | 17:54 | 00:04 | 0 | No | NA |
| 4 | Low wolf | No playback | 26/09/2020 | 18:15 | 17:45 | 17:17 | NA | 0 | Yes | Emerged too early |
| 6 | Low wolf | Wolf | 10/09/2020 | 18:53 | 18:23 | 19:30 | 01:07 | 0 | No | NA |
| 6 | Low wolf | No playback | 12/09/2020 | 18:49 | 18:19 | 19:10 | 00:51 | 1 | No | NA |
| 6 | Low wolf | Wolf | 13/09/2020 | 18:46 | 18:16 | 19:24 | 01:08 | 1 | No | NA |
| 6 | Low wolf | No playback | 14/09/2020 | 18:44 | 18:14 | 19:20 | 01:06 | 1 | No | NA |
| 6 | Low wolf | No playback | 15/09/2020 | 18:41 | 18:11 | 19:04 | 00:53 | 1 | No | NA |
| 6 | High control | Control | 16/09/2020 | 18:39 | 18:09 | 19:11 | 01:02 | 1 | No | NA |
| 6 | High control | Control | 17/09/2020 | 18:37 | 18:07 | 19:13 | 01:06 | 1 | No | NA |
| 6 | High control | Control | 18/09/2020 | 18:34 | 18:04 | 19:22 | 01:18 | 1 | No | NA |
| 6 | High control | Control | 19/09/2020 | 18:32 | 18:02 | 19:27 | 01:25 | 1 | No | NA |
| 6 | High control | Control | 20/09/2020 | 18:29 | 17:59 | 19:07 | 01:08 | 1 | No | NA |
| 6 | High control | Control | 21/09/2020 | 18:27 | 17:57 | 19:09 | 01:12 | 1 | No | NA |
| 6 | High wolf | Wolf | 22/09/2020 | 18:24 | 17:54 | 19:21 | 01:27 | 1 | No | NA |
| 6 | High wolf | Wolf | 23/09/2020 | 18:22 | 17:52 | 19:06 | 01:14 | 1 | No | NA |
| 6 | High wolf | Wolf | 24/09/2020 | 18:20 | 17:50 | 19:51 | 02:01 | 1 | No | NA |
| 6 | High wolf | Wolf | 25/09/2020 | 18:17 | 17:47 | 18:55 | 01:08 | 1 | No | NA |
| 6 | High wolf | Wolf | 26/09/2020 | 18:15 | 17:45 | 19:08 | 01:23 | 1 | No | NA |
| 6 | High wolf | Wolf | 27/09/2020 | 18:12 | 17:42 | 19:01 | 01:19 | 1 | No | NA |
| 6 | Low control | Control | 28/09/2020 | 18:10 | 17:40 | 19:07 | 01:27 | 1 | No | NA |
| 6 | Low control | No playback | 29/09/2020 | 18:08 | 17:38 | 18:41 | 01:03 | 1 | No | NA |
| 6 | Low control | No playback | 30/09/2020 | 18:05 | 17:35 | 19:05 | 01:30 | 1 | No | NA |
| 6 | Low control | Control | 01/10/2020 | 18:03 | 17:33 | 18:45 | 01:12 | 1 | No | NA |
| 6 | Low control | No playback | 02/10/2020 | 18:01 | 17:31 | 18:33 | 01:02 | 1 | No | NA |
| 6 | Low control | No playback | 03/10/2020 | 17:58 | 17:28 | 18:36 | 01:08 | 1 | No | NA |
| 7 | Low wolf | No playback | 16/09/2020 | 18:39 | 18:09 | 19:12 | 01:03 | 0 | No | NA |
| 7 | Low wolf | No playback | 19/09/2020 | 18:32 | 18:02 | 19:05 | 01:03 | 1 | No | NA |
| 7 | Low wolf | No playback | 20/09/2020 | 18:29 | 17:59 | 19:05 | 01:06 | 0 | No | NA |
| 8 | Low wolf | No playback | 07/09/2020 | 19:00 | 18:30 | 18:50 | 00:20 | 0 | No | NA |
| 9 | High control | Control | 30/09/2020 | 18:05 | 17:35 | 18:21 | 00:46 | 0 | No | NA |
| 0 | Low control | No playback | 17/09/2020 | 18:37 | 18:07 | 19:03 | 00:56 | 0 | No | NA |

**Table S2** The average emergence time and next day sett use by playback type at each sett.

| Sett # | Playback type | Emergence time (min) | Next day sett use (1 = presence, 0 = absence) |
| --- | --- | --- | --- |
| 1 | Control | 54 | 0.71 |
| 2 | Control | 57 | 0.50 |
| 3 | Control | 95 | 1.00 |
| 4 | Control | 4 | 0.00 |
| 6 | Control | 74 | 1.00 |
| 9 | Control | 46 | 0.00 |
| 1 | No playback | 41 | 0.50 |
| 2 | No playback | 74 | 0.50 |
| 3 | No playback | 46 | 0.00 |
| 6 | No playback | 65 | 1.00 |
| 7 | No playback | 65 | 0.33 |
| 8 | No playback | 20 | 0.00 |
| 0 | No playback | 56 | 0.00 |
| 1 | Wolf | 58 | 0.83 |
| 2 | Wolf | 71 | 0.50 |
| 3 | Wolf | 29 | 0.50 |
| 4 | Wolf | 33 | 0.00 |
| 6 | Wolf | 81 | 0.88 |

**Table S3** Parameter estimates for the linear mixed model describing badger emergence times.

| **Parameters** | **Estimate** | **SE** | **T** | **P** |
| --- | --- | --- | --- | --- |
| Intercept | 55.595 | 7.002 | 7.940 | < 0.001* |
| No playback | -7.906 | 5.482 | -1.442 | 0.156 |
| Wolf playback | 1.767 | 4.905 | 0.360 | 0.720 |
| Day of year | 5.429 | 2.228 | 2.436 | 0.019* |

**Table S4** Parameter estimates for the generalised binomial mixed model describing badger next day sett use.

| **Parameters** | **Estimate** | **SE** | **Z** | **P** |
| --- | --- | --- | --- | --- |
| Intercept | 0.4493 | 1.001 | 0.446 | 0.655 |
| No playback | -1.178 | 0.986 | -1.195 | 0.232 |
| Wolf playback | -0.650 | 0.913 | -0.713 | 0.476 |

**Figure S1** The number of days badgers were recorded at each sett per playback type. In total, over the field study period each sett was subject to wolf playbacks on 16 days, control playbacks on 16 days, and no playbacks on 8 days.

**Figure S2** Model diagnostic plots for the linear mixed effects model describing emergence time.

**Figure S3** Model diagnostic plots for the generalised binomial mixed model describing next day sett use.

**Figure S4** The 5 setts for which we obtained responses to wolf playbacks overlaid with the landscape level gradient in wolf encounter rates, from light orange (lowest rates) to dark red (highest rates). Figure was included to show that the 5 setts were poorly distributed along the landscape level wolf risk gradient, which precluded our being able to explore the interactive effects of wolf playbacks (risky times) and the landscape level perceived wolf risk gradient (risky places) on badger denning behaviour. Sett numbering corresponds to the numbering in Tables S1 and S2. Wolf encounter rates refer to the patterns of space use of wolves in 500 × 500 m grid cells (as determined by camera trapping in Bubnicki et al. 2019). We generated the image in QGIS v 3.16.16 610 (www.qgis.org).
